# Geckos cling best to, and prefer to use, rough surfaces

**DOI:** 10.1186/s12983-020-00374-w

**Published:** 2020-10-16

**Authors:** Rishab Pillai, Eric Nordberg, Jendrian Riedel, Lin Schwarzkopf

**Affiliations:** College of Science and Engineering, James Cook University, Townsville, QLD 4810 Australia

**Keywords:** Adaptation, Attachment, *Oedura*, Performance, Peak-to-valley height, Substrates, Shear force

## Abstract

**Background:**

Fitness is strongly related to locomotor performance, which can determine success in foraging, mating, and other critical activities. Locomotor performance on different substrates is likely to require different abilities, so we expect alignment between species’ locomotor performance and the habitats they use in nature. In addition, we expect behaviour to enhance performance, such that animals will use substrates on which they perform well.

**Methods:**

We examined the associations between habitat selection and performance in three species of *Oedura* geckos, including two specialists, (one arboreal, and one saxicolous), and one generalist species, which used both rocks and trees. First, we described their microhabitat use in nature (tree and rock type) for these species, examined the surface roughnesses they encountered, and selected materials with comparable surface microtopographies (roughness measured as peak-to-valley heights) to use as substrates in lab experiments quantifying behavioural substrate preferences and clinging performance.

**Results:**

The three *Oedura* species occupied different ecological niches and used different microhabitats in nature, and the two specialist species used a narrower range of surface roughnesses compared to the generalist. In the lab, *Oedura* geckos preferred substrates (coarse sandpaper) with roughness characteristics similar to substrates they use in nature. Further, all three species exhibited greater clinging performance on preferred (coarse sandpaper) substrates, although the generalist used fine substrates in nature and had good performance capabilities on fine substrates as well.

**Conclusion:**

We found a relationship between habitat use and performance, such that geckos selected microhabitats on which their performance was high. In addition, our findings highlight the extensive variation in surface roughnesses that occur in nature, both among and within microhabitats.

## Background

Habitat use has been a critical variable included in ecological niche studies for several decades [1]. A common functional requirement for successful niche use is effective locomotion within the environment (particularly on specific microhabitats, for example on trees or rocks). Locomotory ability influences an animal’s success at capturing prey, avoiding predators, and acquiring mates [2–5], thereby influencing growth rates, survival, reproduction, and consequently, Darwinian fitness [3, 6–8]. If variation in performance in relation to substrate microhabitat use is adaptive, species should use substrates that enhance locomotor capabilities to increase fitness in nature [9–11]. Given that species exploit a range of topographical features within their environment, we expect natural selection to act on locomotor performance in relation to the specific challenges encountered [9, 12, 13]. Examples of relationships between performance and habitat use exist in a range of habitats, including marine [14], aerial [15], arboreal (e.g., *Anolis*, [11, 16, 17]), and terrestrial (e.g., *Tropidurus* sp. [14, 18]; Lacertids, [19]; and skinks, [20, 21]) environments. In general, this work suggests the presence of trade-offs, in which adaptations that optimize performance in certain habitats may not be beneficial in others [8]. For example, traits like foraging performance [20], running [19], sprinting [18, 19], and clinging [19, 21] are a function of morphological adaptations that evolve in the context of habitat. These relationships between habitat use and performance, may mean animals only use certain subsets of the entire range of microhabitats available, in which their performance is high, or may, at least, avoid microhabitats in which they do not perform well. Therefore, it is essential to incorporate habitat selection behaviour into studies investigating the relationship between habitat structure and performance [8].

Both the general nature of the habitat (e.g., open versus closed habitats [9, 18, 22]) and specific structural components like incline [23, 24], and perch characteristics [8, 25–27] influence performance capabilities. Locomotor performance shapes habitat use [14], such that species that can move well on a range of substrate widths, for example, occupy a broader range of habitats than species specialized to narrow range of substrates [8, 26]. Further, rocky habitats appear to select for increased jumping capacity and lower absolute sprint speeds [12], increased limb lengths [19], and resistance to mechanical traction [21], compared to sandy [16, 21, 28] and other habitats [19, 21]. Recently, some studies have investigated performance in relation to structural components like surface textures and microtopographies [29–36]. Microtopographical characteristics like surface roughness [29–32, 34, 35], fouling [30] and periodical wrinkles [33] affect attachment capabilities of various taxa, which influence performance due to differences in the area available for contact with the attachment apparatus [10, 37, 38]. Attachment capabilities of adhesive systems, are generally higher on smooth surfaces that provide a greater area for attachment as compared to rough surfaces [31, 34]. Studies examining the range of morphologies and capabilities on natural surfaces remain rare in some systems, but are critical to understanding selective forces.

Geckos (Gekkota) are a diverse and widely distributed lizard clade comprising of approximately 2008 species of geckos belonging to over 100 genera [39]. Geckos occupy a variety of habitats [40–43], and are well known for their specialized adhesive toepads and climbing abilities [44, 45]. The development of adhesive toepads has enabled geckos to use inverted and inclined surfaces on rocks and vegetation [43, 46, 47]. Adhesion occurs through finely tuned, hierarchically arranged adhesive toe pads [44, 48–50], characterized by subdigital scansors that carry highly organized fields of microfibrillar setae. Each seta is branched, and branches terminate in broadened tips called spatulae [49, 51, 52]. Friction is achieved as van der Waals forces generating a normal force between the spatulae and the substrate [44, 53]. Substrate surface topology influences the area available for attachment of the setae, and high surface area increases the magnitude of force generated [52]. Thus, the adhesive system of geckos was thought to perform better on smooth and uniform surfaces [10]. Recently, however, some studies have found that geckos are capable of attachment on rough and undulant substrates, previously thought to provide limited purchase for attachment [10, 54]. Clearly, further study of gecko performance on rough and undulant surfaces, similar to those geckos encounter in nature, is required [10, 11, 38, 45, 54–56].

Studying functional relationships between performance and texture (microtopography) is important for understanding the biomechanics of gecko adhesion, but to understand the evolutionary, adaptive, and ecological aspects of surface texture and its relationship with fitness, we need to understand how geckos use surfaces they encounter in the wild. We investigated microhabitat choice in the context of locomotory performance, both in the laboratory and in nature, in three sympatric species of the Australian Diplodactylid gecko genus *Oedura*: one saxicoline or rock-dwelling species, one arboreal species, and one generalist species that uses both rocks and trees (this study, [41, 45]). We quantified habitat choice of these geckos in the field, and measured roughness (as peak-to-valley heights, a two-dimensional measure of surface roughness), of surfaces used by these geckos in nature. We then brought individuals of all three species into the laboratory, and examined their microhabitat choice using artificial substrates that spanned the surface roughnesses we measured in nature. Finally, we measured shear force as a measure of performance on these different artificial substrates, to determine if there was a link between substrate choice and performance [21, 37, 42]. We hypothesized that geckos select substrates, and would perform better on surfaces more similar to those they use in nature. We also hypothesized that, if gecko performance was closely adapted to the substrates they used, the generalist species may perform better (on average) on a variety of microhabitats, whereas specialists may be more likely to exhibit better performance on specific habitats.

## Results

### Microhabitat use in nature

**Fig. 1:**
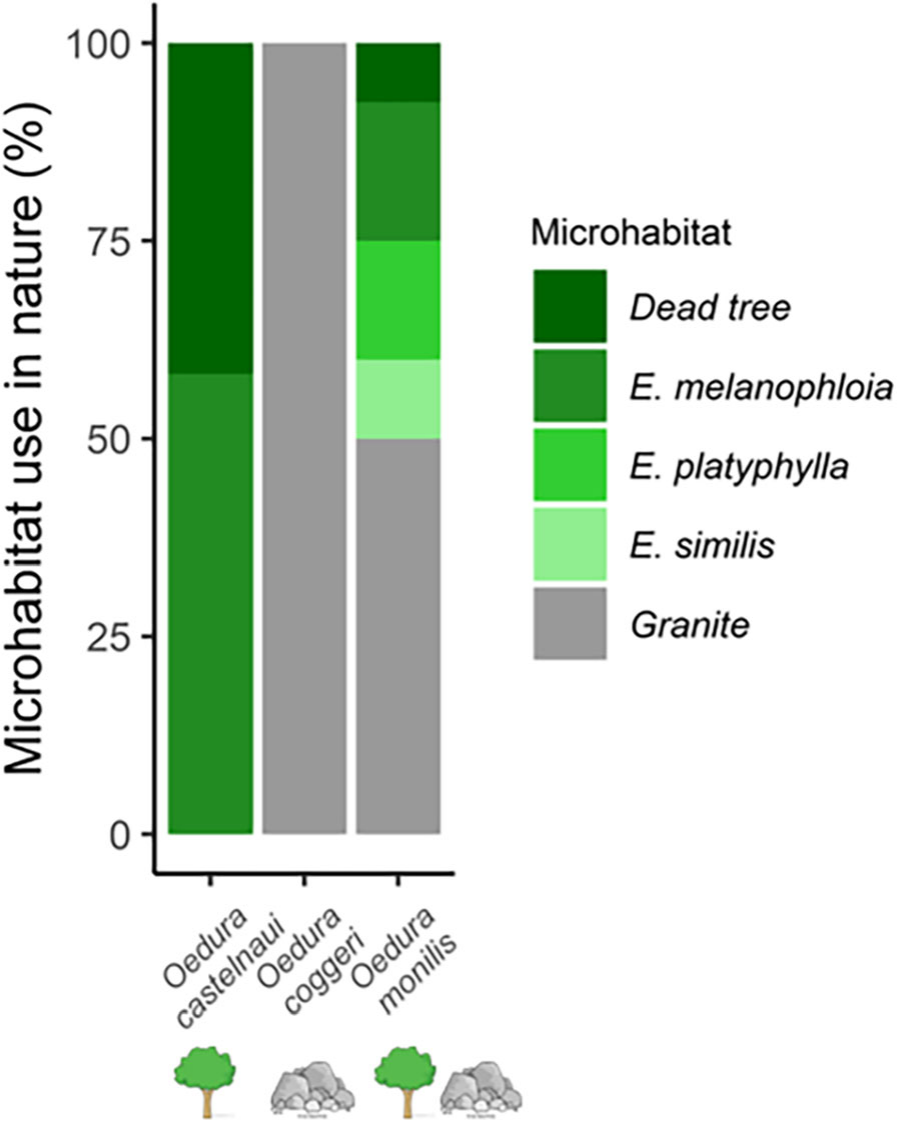
Microhabitat use in *Oedura* geckos (%). Substrates were: dead trees, silver-leaved ironbark (*E melanophloia)*, Cabbage Gum (*E. platyphylla)*, Queensland yellowjacket paperbark (*E. similis)* (shades of green) and granite (grey). Amounts shown are the percentage of observations (*O. castelnaui*, *n* = 67; *O. monilis*, *n* = 40; and *O. coggeri*, *n* = 17, total 124 observations) on tree and rock types within their habitats

**Fig. 2:**
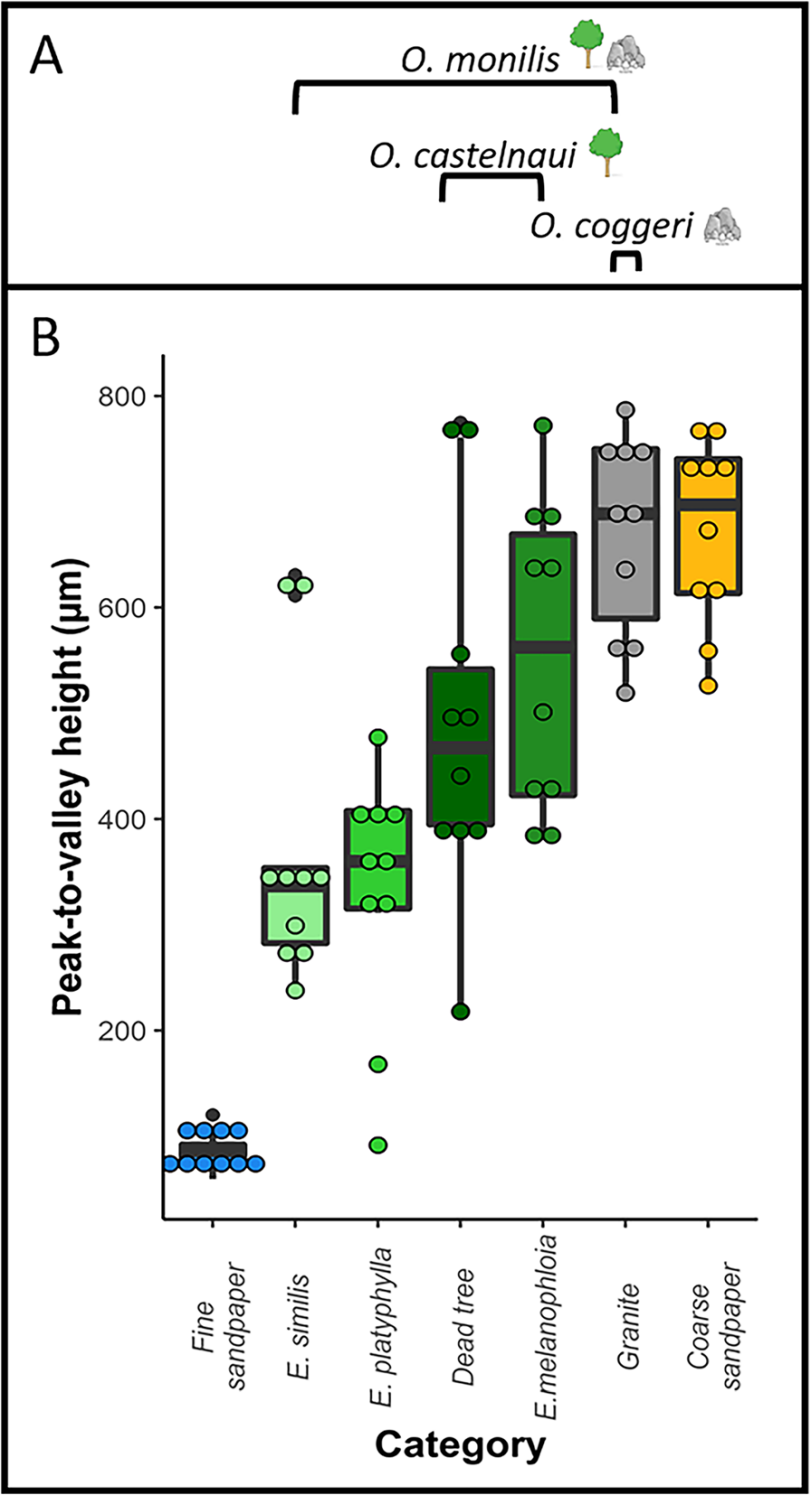
**a** Black brackets encompass substrates used by three *Oedura* geckos: saxicoline, *O. coggeri* (granite), arboreal *O. castelnaui* (dead trees and *Eucalyptus melanophloia*) and *O. monilis* (*E. similis*, *E. platyphylla*, dead trees, *E. melanophloia* and granite). **b** Peak-to-valley heights of natural and test substrates (μm). Natural substrates include *E. similis*, *E. platyphylla*, dead trees, *E. melanophloia* (shades of green) and granite (grey). Test substrates consisted of coarse sandpaper (P40 grit; blue) and fine sandpaper (P400 grit; orange)

The habitat use we observed corresponded well to habitat use reported in the literature for these species, according to which northern velvet geckos (*O. castelnaui*) use arboreal habitats [42, 55, 57]; spotted velvet geckos (*O. coggeri*) use saxicoline habitats, [42, 54]; and ocellated velvet geckos (*O. monilis*) use both arboreal and saxicoline habitats [42, 47, 58]. We found *Oedura castelnaui* (*N* = 67) exclusively on arboreal microhabitats and used dead trees and silver-leaf ironbark trees (*Eucalyptus melanophloia)* approximately equally (Fig. 1). *Oedura monilis* (*N* = 40) were found in approximately equal proportions on both arboreal and saxicoline habitats, arboreal habitats included dead trees, *E. melanophloia*, cabbage gums (*E. platyphylla)*, paperbark trees (*E. similis),* and saxicoline habitats were comprised of granite (Fig. 1). *Oedura coggeri* (*N* = 17) were found exclusively on granite (Fig. 1). Furthermore, the generalist species (*O. monilis*) encountered a wider range of surface roughness compared to the arboreal and saxicolous species (Fig. 2a, Supplementary material S1).

### Microhabitat roughness and selection of test substrates

Peak-to-valley heights are a two-dimensional measure of surface roughness used to compare surface microtopographies in this study. Natural substrates that formed the microhabitats used by *Oedura* geckos exhibited significantly different peak-to-valley heights (Kruskal-Wallis rank sum test, χ^2^ = 48.64, df = 6, *p*-value < 0.01). Paperbark trees (*E. similis,* Mean peak-to-valley height ± SE = 370.20 ± 27.77 μm) exhibited similar peak-to-valley heights to cabbage gums (*E. platyphylla,* 331.74 ± 37.32 μm, Wilcoxon rank sum test, *P* = 0.77) and dead trees (491.50 ± 54.29 μm, Wilcoxon rank sum test, *P* = 0.07). *Eucalyptus similis* exhibited significantly lower peak-to-valley heights compared to silver-leaf ironbark trees (*E. melanophloia,* 554.60 ± 45.83 μm, Wilcoxon rank sum test, *P* < 0.01) and granite (668.90 ± 30.00 μm, Wilcoxon rank sum test, *P* < 0.01). Granite exhibited similar peak-to-valley heights to ironbark trees (*E. melanophloia,* Wilcoxon rank sum test, *P* = 0.09), however, exhibited significantly higher peak-to-valley heights than dead trees (Wilcoxon rank sum test, *P* < 0.05), cabbage gums (*E. platyphylla,* Wilcoxon rank sum test, *P* < 0.01) and paperbarks (*E. similis,* Wilcoxon rank sum test, *P* < 0.01; Fig. 2b).

Among our test substrates, peak-to-valley heights of fine sandpaper (P400 grit, 85.76 ± 5.36 μm) were similar in roughness to the very smoothest tree bark we measured, and were significantly different from all natural substrates (Wilcoxon rank sum test, *P* < 0.01), and to the coarse sandpaper (P40 grit, 672 ± 27.77 μm, Wilcoxon rank sum test; *P* < 0.001) used in our study. Coarse sandpaper used in our study had peak-to-valley heights not significantly different from silver-leaf ironbark (*E. melanophloia,* Wilcoxon rank sum test, *P* = 0.12) and granite (Wilcoxon rank sum test, *P* = 1.00; Fig. 2b).

### Assessment of microhabitat choice in the laboratory

**Fig. 3:**
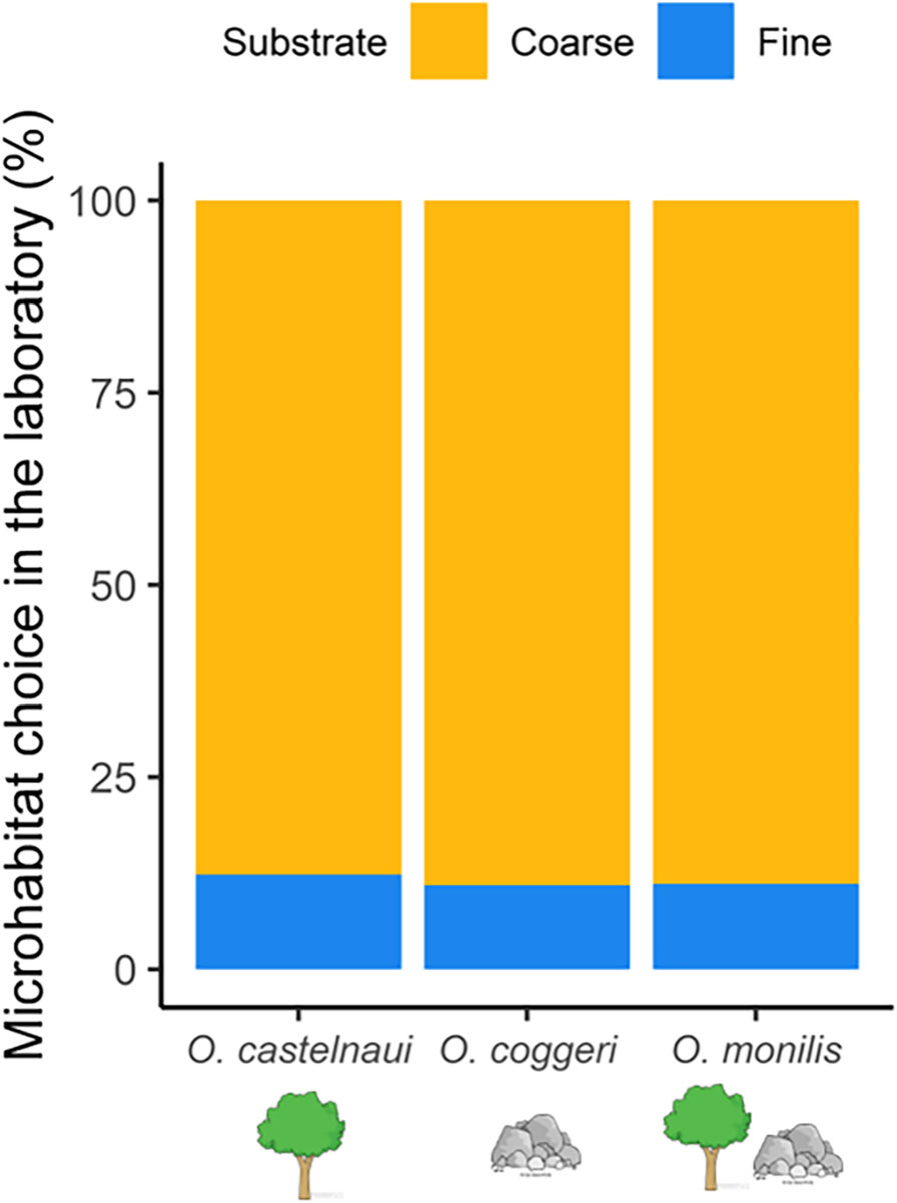
Microhabitat choice of *Oedura* geckos in the laboratory (%). Mean percent of observations (*n* = 10 individuals of each species), amounts shown are the mean proportion of observations (range = 0 to 91) on each substrate for three *Oedura* species. Substrates were coarse sandpaper (P40 grit; orange) and fine sandpaper (P400 grit; blue)

The best model (ΔAICc < 2) predicting microhabitat choice (in the laboratory) included only ‘substrate’ as a fixed effect and individual gecko ID as a random factor (marginal *R*^*2*^ = 0.57, conditional *R*^*2*^ = 0.57, Table 1 and Fig. 3), indicating that substrate choice did not vary significantly among species. Repeating analyses including use of both vertical and horizontal surfaces did not affect our conclusions about microhabitat choice in the laboratory (marginal *R*^*2*^ = 0.05, conditional *R*^*2*^ = 0.06, Supplementary material S2).

**Table 1:** Models included in selection using Akaike’s information criterion to analyse microhabitat choice in *Oedura* geckos, *O. castelnaui, O. monilis* and *O. coggeri.* Models are arranged in increasing order of ΔAIC values and top models are in bold

Fixed effects	ΔAIC	df	Weight	Residual Deviance
**Substrate**	**0.0**	**3**	**0.85**	**617.9**
Substrate + Species	4.0	5	0.12	617.9
Substrate*Species	6.2	7	0.04	616.1
Species	1807.63	4	< 0.001	2423.2

*Abbreviation*: *df* degrees of freedom

All three gecko species spent a significantly more time on coarse sandpaper (mean proportion of observations ± SE = 0.88 ± 0.008) compared to fine sandpaper (0.11 ± 0.008; Fig. 3). The coarse sandpaper had peak-to-valley heights similar to ironbark (*E. melanophloia)* and granite, whereas the fine sandpaper they used less had peak-to-valley heights similar to the lowest measurement for any substrate we made in nature (cabbage gum *E. platyphylla* extremes*;* Fig. 2).

### Shear force as a measure of clinging performance

**Fig. 4:**
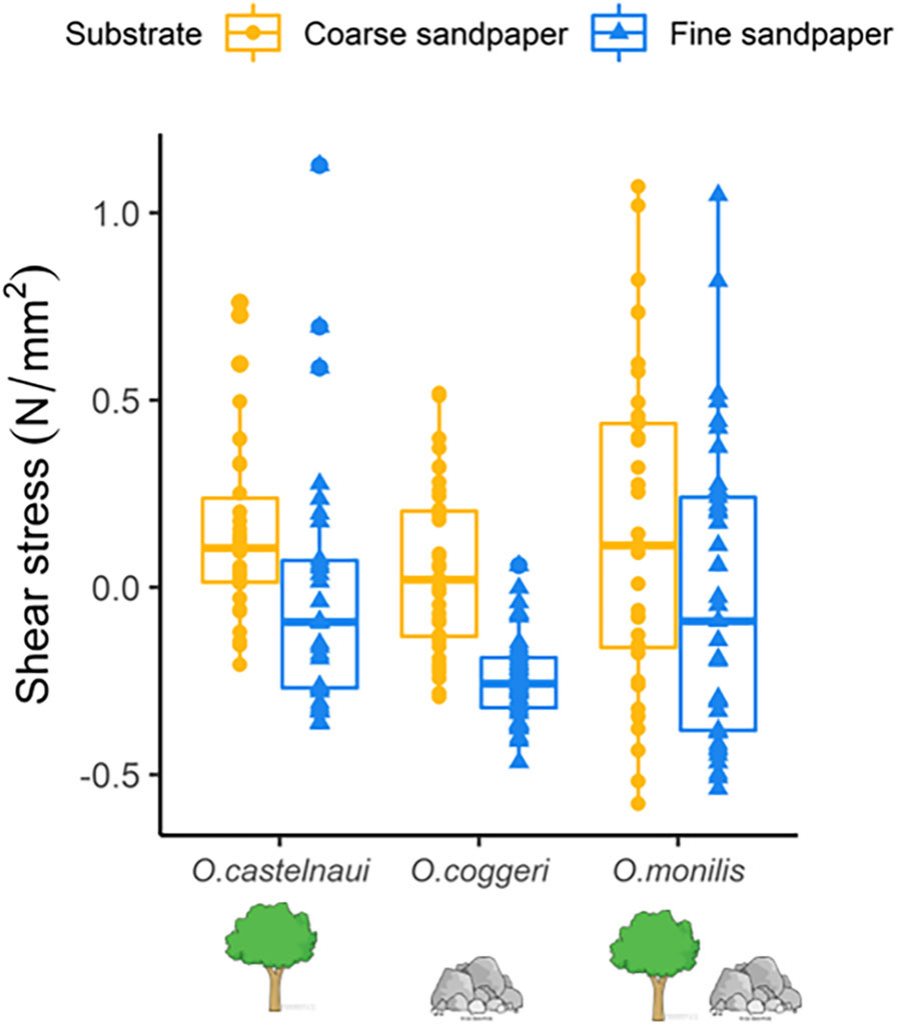
Clinging performance of *Oedura* geckos (N/mm^2^); three measures per substrate for 24 individuals and five measures per substrate for eight individuals on test substrates. Measures shown are shear stress (residuals of regressing absolute force against toe pad area) by *O. castelanui* (*N* = 10), *O. coggeri* (*N* = 11) and *O. monilis* (*N* = 11) on coarse sandpaper (P40 grit; orange) and fine sandpaper (P400 grit; blue). Dots represent each shear force measure (Newtons) for each individual on coarse sandpaper. Triangles represent shear force measures (Newtons) for each individual on fine sandpaper

Both models with a ΔAIC < 2 included a species-by-substrate interaction, and mass, while one of these two models also included toepad area (Table 2). Post hoc analysis on the models including the variables of high relative importance showed that shear force exerted by the arboreal species (*O. castelnaui, N* = 10) was greater on coarse sandpaper (mean ± SE, 0.59 ± 0.07 N) compared to fine sandpaper (0.39 ± 0.04 N; estimated marginal least square means post hoc comparison: df = 223, t = 4.43, *P* < 0.001, Fig. 4). Similarly, the saxicolous species (*O. coggeri, N* = 11) also exerted greater shear force on coarse sandpaper (0.70 ± 0.07 N) compared to fine sandpaper (0.37 ± 0.04 N; estimated marginal least square means post hoc comparison: df = 223, t = 6.83, *P* < 0.001). Clinging performance was not significantly different on coarse sandpaper (0.69 ± 0.07 N) compared to fine sandpaper (0.52 ± 0.06 N) in the generalist species, *O. monilis* (*N* = 11, estimated marginal least square means post hoc comparison: df = 223, t = 2.70, *P* = 0.08; Fig. 4 and Supplementary material S3).

**Table 2:** Models included in selection using Akaike’s information criterion to analyse clinging performance in *Oedura* geckos, *O. castelnaui*, *O. monilis* and *O. coggeri.* Models are arranged in increasing order of ΔAIC values and top models are in bold

Fixed effects	ΔAIC	df	Weight	Residual Deviance
Substrate*Species + log(mass) + log(toepad area)	**0**	**10**	**0.509**	**269.6**
Substrate*Species + log(mass)	**1.2**	**9**	**0.273**	**269.6**
Substrate + log(mass) + log(toepad area)	3.5	8	0.090	273.8
Substrate + Species + log(mass) + log(toepad area)	3.7	6	0.079	278.0
Substrate + Species + log(mass)	4.7	7	0.049	277.0
Substrate*Species + log(toepad area)	19.5	9	< 0.001	287.8
Substrate + Species + log(toepad area)	22.1	7	< 0.001	294.5
Substrate + Species + log(toepad area) + log(mass)	27.2	5	< 0.001	303.6
Substrate + log(toepad area)	33.4	5	< 0.001	309.7
Species + log(mass) + log(toepad area)	55.5	7	< 0.001	327.8
Species + log(mass)	56.3	6	< 0.001	330.6
Species + log(toepad area)	68.1	6	< 0.001	342.4

*Abbreviation*: *df* degrees of freedom

## Discussion

Our measurements of habitat use were consistent with literature reports for these species [42, 47, 54, 57], but we report the range of roughnesses used by these geckos in nature (Fig. 1). In nature, the saxicoline species used the narrowest range of roughnesses, the arboreal species a slightly wider range, and the generalist the widest range (Fig. 2a and Supplementary material S1). The coarse sandpaper (P40grit; 672 ± 27.77 μm) we used as a test substrate in our experiments, exhibited peak-to-valley heights similar to the roughest surfaces used by these geckos in nature, silver-leaf ironbark (*E. melanophloia;* 554 ± 45.83 μm) and granite (668.90 ± 30.00 μm), whereas the fine sandpaper (P400; 85.76 ± 5.36 μm) exhibited peak-to-valley heights comparable to the least rough substrates used in nature (some measures of cabbage gum bark, *E. platyphylla;* 331.74 ± 37.32 μm; Fig. 2). Consistent with the roughness of many of the substrates they used in nature, all three species preferred coarse sandpaper in laboratory assessments of microhabitat choice (Fig. 3). In addition, clinging performance in the arboreal and saxicolous species was greater on coarse than on fine sandpaper, and thus the microhabitats they selected in the laboratory were those on which clinging performance was high. Interestingly, the generalist species also preferred coarse sandpaper, although its clinging performance was only slightly, and not significantly, higher on coarse sandpaper (Fig. 4). Thus, although the generalist species in our study preferred rougher substrates in the laboratory, their ability to cling did not vary significantly between test substrates, suggesting that their adhesive system is capable of competent attachment to a wider range of substrates than the microhabitat specialists. Thus, our findings were consistent with the hypothesis that species should select substrates on which they perform well. In addition, we provide important considerations for studies investigating gecko attachment systems in relation to ecology and specifically, surface roughness.

### Microhabitat roughness and selection of test substrates

Locomotory capabilities are a consequence of mechanical interactions between animals and the substratum, and the attachment capability of geckos is one such surface interaction [56, 59, 60]. Over the past few decades, studies investigating locomotor performance have mostly focused on very smooth substrates [10, 44, 54, 61, 62], however, recently some studies have used naturally rough and ecologically relevant substrates [10, 38, 62–64]. We used artificial surfaces (sandpaper) for uniformity, but selected roughnesses (measured as peak-to-valley heights) similar to those of natural substrates (among the roughest and least rough) used by our geckos in nature. Our findings highlight the variation that exists within and among substrates used by geckos in nature, and were consistent with previous studies showing there was high variation in rock surface roughnesses used by geckos [10, 54]. Our three gecko species faced broadly similar challenges, at least in terms of roughness. The granite (668.9 ± 67.88 μm) used by our saxicolous species had average roughness similar to the roughest trees (*E. melanophloia;* 554.60 ± 45.83 μm; Wilcoxon rank sum test, *P* = 0.09) frequently used by the arboreal and generalist species in our study, although on average, species using arboreal environments experienced a range of roughnesses, as did the generalist, which experienced the widest range. Thus, classifying species only as ‘arboreal’ or ‘saxicoline’ might not be informative to predict required performance, instead, perhaps more details of surface microtopography should be examined.

The need to incorporate an ecological component into gecko performance studies, especially surface roughness, has been highlighted recently [61, 65]. Although peak-to-valley heights are one important measure expressing characteristics of surface microtopography, they do not consider other surface characteristics, such as shape and spacing of asperities, which also could influence shear forces generated by geckos [56, 60, 62]. Future studies should incorporate other, potentially more informative, three-dimensional measurements of roughness produced from multiple two-dimensional profiles [10, 62, 66]. Furthermore, fabricating particular surface characteristics, while standardising all other properties, would allow us to accurately assess performance exclusively in the context of surface microtopography [10, 62].

### Assessment of microhabitat choice in the laboratory

One ecological factor influencing locomotor performance is microhabitat use [38], as different substrates will impose different requirements for performance [17], making habitat selection a critical determinant of the relationship between performance and fitness [8]. Laboratory comparisons of functional capabilities may be meaningful only if comparable habitats are used in the field [8]. *Oedura* geckos preferred coarse sandpaper (P40grit; 672 ± 27.77 μm) in our study, which had microtopography in some ways similar to granite (668.90 ± 30.00 μm; Wilcoxon rank sum test, *P* = 1.00) and ironbark (*E. melanophloia;* 554 ± 45.83 μm; Wilcoxon rank sum test, *P* = 0.12) used in nature (Figs. 2 and 3), suggesting our study was at least moderately relevant to substrate choice in nature [8], although further comparisons, for example, using samples of natural materials, would be informative.

A range of factors, such as temperature [55, 67], substrate colour and height [68], ambient light [69, 70], competition [9, 57] and refuge availability [70] influence microhabitat selection in geckos. We excluded the influence of these confounding factors by performing trials in constant temperature rooms, matching substrate colour, using infrared light, and not providing any refuge in the testing arenas (Supplementary material S5). An advantage of our experimental design was that surface microtopography was the only characteristic that varied between the sandpaper types used in this study, but we were forced to use artificial substrates to achieve this level of control. Using natural substrates also has advantages, but introduces a range of variables other than surface microtopography into habitat choice. Experiments using both approaches will help us better understand the relationship between performance and habitat choice in these animals.

### Shear force as a measure of clinging performance

In general, habitat specialists use a narrow spectrum of available habitats, and are expected to perform better in these specific habitats compared to generalists [11, 20, 71–73]. For specialists, broader habitat use is thought to entail a trade-off, in which they cannot perform as well in other habitats compared to those in which they specialise [20, 71], whereas generalists are thought to have lower overall performance, but to perform better in various habitats (the ‘jack of all trades is master of none’ concept). Thus, we expected the generalist, *O. monilis*, to have the capacity to perform well on a range of microhabitats, and, for the same reasons, we did not expect better performance on either substrate type [62]. Consistent with this, clinging performance of *O. monilis* was highly variable and was not significantly different between coarse and fine sandpaper (Fig. 4). Surprisingly, the performance of *O. monilis* was not worse than the specialists on the rougher sandpaper. Possibly, morphological adaptations in some geckos can permit attachment to a wide variety of unpredictable surfaces, instead of being specially adapted to specific substrate types [10]. If all gecko attachment systems have evolved to attach to the range of substrates they may encounter in nature, then we would not expect increased clinging performance on subsets of microhabitats, but we did observe this in the rock and tree specialists. On the other hand, perhaps the need to attach to a range of different surfaces simply selects for high performance on that range of substrates, without incurring trade-offs.

The attachment mechanisms of geckos are certainly efficient on very smooth artificial substrates (e.g., glass, acetate, Plexiglass and polishing film), and can diminish on rough or undulant natural surfaces [74]. This may be because smooth surfaces generally provide more surface area available for contact [10, 38]. Preliminary experiments in our laboratory, blocking setal fields (pers obs.), indicate that our observations of high performance on coarse and undulant substrates is a function of both claws and setal fields, claws alone impart significantly lower shear forces. Given that these geckos have both claws and setae, we expect clinging performance to be high on substrates that provide purchase for both components to attach [54, 75, 76]. Furthermore, specific morphology of both claws and toes can increase performance on rough substrates [77]. All three *Oedura* species exhibited good clinging performance on rough substrates, therefore, future studies should examine both claw and toe morphology in relation to substrates they encounter and use in nature.

## Conclusion

Our study examined whether clinging performance, in the context of substrate roughness, is related to microhabitat preference. We found that surface roughness of natural substrates was highly variable, highlighting complexity even within specific habitat categories (i.e., ‘trees’). Therefore, future studies should ensure classifications of gecko habitats provide sufficient detail to describe surface microtopographies encountered by geckos in nature. All three *Oedura* geckos preferred coarse sandpaper, upon which they performed best, and which was similar in roughness to the substrates they encountered and used in nature. The shear forces imparted by the generalist were high on both substrates, consistent with their use of both coarse- and fine-grained surfaces in nature, and they preferred coarse sandpaper in the lab. Our findings revealed an association between habitat preference and performance, and provide further evidence for the capacity of the gecko adhesive system to accommodate a wide range of surface roughness.

## Methods

We assessed microhabitat use in *Oedura* geckos by compiling observations from individuals sighted or captured in nature (124 observations). To assess surface roughness used by these geckos, and to determine the roughnesses of the surfaces used in the lab in relation to natural surfaces, we measured the peak-to-valley heights (μm) of the surfaces geckos used, and also measured the two artificial surfaces (sandpaper) we used to assess microhabitat choice (*N* = 10 for the three *Oedura* species) and clinging performance (*O. castelnaui*, *N* = 10; *O. coggeri*, *N* = 11, and *O. monilis*, *N* = 11) in the laboratory. From the geckos observed in nature, a subset of individuals of each species were collected for laboratory trials of microhabitat choice and clinging performance.

### Study species

We studied three closely related [78], and otherwise morphologically similar [79], species of velvet gecko (genus *Oedura*) with well-developed toepads, namely, northern velvet geckos (*Oedura castelnaui*), northern spotted velvet geckos (*Oedura coggeri*) and ocellated velvet geckos (*Oedura monilis*, [41, 53, 55, 77]).

### Microhabitat use in nature

To assess microhabitats used by these geckos, we compiled observations from 2015 to 2020. We recorded microhabitat categories used by geckos (tree species and rock types). We included observations from different times of the year and from a range of localities (Supplementary material S4). We used a total of 124 observations of three species (*O. castelnaui*, *N* = 67; *O. monilis*, *N* = 40; and *O. coggeri*, *N* = 17) and calculated the percent of observations for each species on each microhabitat type.

### Microhabitat roughness and selection of test substrates

All three species occurred in open eucalyptus woodlands, but used different microhabitats. We measured the peak-to-valley heights of substrates used by our geckos using a surface profile gauge (Landtek Srt-6223, Accuracy: ± 5 μm; Resolution: 0.1 μm/1 μm). Ten randomly selected points on each substrate were measured to quantify the variation in surface roughness encountered by these geckos in nature. We compared the roughness of all substrates with non-parametric tests (substrate roughnesses were not normally distributed) using a Kruskal-Wallis Test, followed by Wilcoxon Signed Rank tests for post hoc comparisons of substrate roughness.

For our laboratory assessments of microhabitat choice and clinging performance, we wanted substrates with a quantifiable range of roughnesses similar to the ranges found in nature, but with uniform surface chemistry, so we used sandpaper exhibiting peak-to-valley heights representing the highest and lowest measures of peak-to-valley heights used by our geckos in nature.

### Field collection

To use in laboratory trials, adult geckos of each species were collected by hand during spotlighting surveys in northeast Queensland, Australia between June–August 2016. The arboreal species (*O. castelnaui;* 4:6 male to female) were collected from eucalyptus open woodland habitats on the James Cook University, Townville campus. The generalist species (*O. monilis;* 5:6) and the saxicolous species (*O. coggeri;* 4:7) were collected from open woodlands and rocky outcrops at three sites at Hidden Valley (Supplementary material S4). Geckos were returned to the laboratory at James Cook University in cloth bags.

Following collection, geckos were housed in controlled temperature rooms (mean ± SE: 25 °C ± 1.5) at the James Cook University, Townsville Campus, and exposed to a 12-h light-dark cycle (0600–1800 L; 1800–0600 D). Geckos were housed individually in plastic enclosures (30 × 15 × 9 cm), with a ceramic tile shelter and water *ad libidum*. To allow geckos to thermoregulate, all enclosures were placed on racks, with heat sources that reached 33 °C during the day running under one end, to form a thermal gradient within each enclosure. Geckos were fed live crickets (*Acheta domestica*) dusted with vitamin and calcium powder supplements (Reptivite™), twice weekly.

### Morphometrics

Snout-vent-length (SVL) and mass were measured on the each species (*O. castelnaui*, *N* = 10, *O. coggeri*, *N* = 11, *O. monilis*, *N* = 11) that were collected and housed in the laboratory, using a ruler and a digital scale (SVL in mm ± 0.1 and mass in g ± 0.05). Surface area of the adhesive toepads may influence clinging performance [10, 80–82]. To measure the surface-area of toepads, the subdigital (ventral) aspect of the hands and feet of all individuals collected and housed at James Cook University were photographed through glass against a uniform dark background with a scale in each image. Lightroom CC (Adobe, 2017) was used to adjust the contrast of images to ensure that the emphasis was on the adhesive lamellae. The thresholding feature in ImageJ [60, 83] was then used to select the toepads by saturation, as they contrasted strongly with the rest of the image. Measurements were calibrated using the scale incorporated in every image. Measurements were taken for all five toes on the right hand (manus) and right foot (pes) of all geckos and doubled to calculate total adhesive area for each gecko (Table 3).

**Table 3:** Mean morphometric measurements (mean ± SD) of the three *Oedura* species

Species	n	SVL (cm)	Mass (g)	Toepad area (mm)
Arboreal (*O. castelnaui*)	10	73.43 ± 10.94	12.28 ± 4.79	50.50 ± 10.77
Generalist (*O. monilis*)	11	82.64 ± 5.77	11.35 ± 1.87	79.35 ± 15.35
Saxicolous (*O. coggeri*)	11	70.52 ± 8.98	7.86 ± 0.96	55.10 ± 9.56

### Assessment of microhabitat choice in the laboratory

Enclosure experiments are a powerful tool to study substrate choice in geckos, as variables like substrate availability can be precisely controlled [62, 84]. Substrate selection testing arenas (plastic containers, 70 × 32 × 12 cm; total area – 2448 cm^2^) were lined on all inner surfaces with equal areas (1224 cm^2^ each) of P40 grit (coarse) and P400 grit (fine) aluminium oxide sandpaper (Active Abrasives Pty Ltd., Australia; Supplementary material S5). We investigated substrate choice in the laboratory using the 10 individuals of each species, each sampled once. The front of the testing arenas were covered with transparent plastic film (Clorox Australia Pty Ltd., New South Wales, Australia) sprayed with canola oil (Pascoe’s, Western Australia, Australia) to allow us to observe the geckos in the arena through the transparent film, while simultaneously keeping geckos from walking on the non-sandpaper surface. No food or shelter was available in the testing arenas and light and heat were uniform within the arenas. Geckos were randomly selected and introduced individually to the centre of the arena on the vertical area facing the observer. To reduce the influence of external variables on geckos’ substrate choice, the orientation of substrates (left-hand side or right-hand side) was randomly selected before each trial. Substrate choice was video recorded between 18:00 and 21:00 under infrared lights using a tripod-mounted DCR-SR55 Sony Handycam (Sony Corporation, Tokyo, Japan) in ‘NightShot’ mode. Two to three separate arenas containing one gecko each were recorded simultaneously in each two-hour video. Before use, enclosures were sprayed with 80% ethanol, cleaned, and allowed to air dry completely to remove any scent from previous geckos. No observers were present in the room during trials.

Videos were reviewed [85] and the substrate upon which the geckos were observed was recorded every minute for 90 min. To eliminate possible behavioural effects of introduction to the testing chamber, the first 15 min of each video were discarded. Instances when geckos were in the centre (i.e., the body spanned both substrates) or when geckos attempted to climb the front (non-sandpaper surface) were excluded from analysis (< 5% of all observations). Having touched the oily surface seemed not to influence gecko behaviour, locomotion or substrate choice. Proportion of time spent on each substrate (count of observations on each substrate divided by total number of observations on both substrates) were calculated for each individual and used as a measure of substrate choice for each individual gecko. Toepads have evolved as a mechanism for climbing [19, 77, 86] and the adhesive apparatus may not be deployed during locomotion on horizontal surfaces [63, 87], therefore, observations on horizontal surfaces were excluded from analysis (46.02% of 2137 observations). To ensure that excluding horizontal observations did not affect our conclusions, we repeated our analysis including both vertical and horizontal observations, which did not affect our conclusions.

To investigate microhabitat choice among gecko species, we used generalized linear mixed-effect models (GLMMs) fit to a binomial distribution using the package *lme4* [88]. We used the proportion of time spent on each substrate by each individual gecko as the response variable. Individual gecko IDs were included as a random factor, to account for variation among individuals in the number of observations on vertical substrates. As binomial responses (choice of coarse or fine sandpaper) were expressed as proportions, total observations of each individual on each substrate were included as ‘weights’ in all candidate models. Four candidate models were constructed with fixed effects as (1) species and substrate, (2) species only, (3) substrate only and (4) species * substrate interaction (Table 4). We identified a best fit model using Akaike’s information criterion (ΔAICc < 2) using the R package *AICcmodavg* [89]. Further, we conducted post hoc pairwise comparisons using the package *emmeans* [90] on the variables included in the best fit model(s). All analyses were carried out in the R program environment [91].

**Table 4:** Four candidate models used to analyse the proportion of time spent on coarse and fine sandpaper in three *Oedura* geckos, *O. castelnaui*, *O. monilis* and *O. coggeri*

Model	Response variable	Random effect	Fixed effect
1	proportion of observations on each substrate	gecko id	substrate + species
2	proportion of observations on each substrate	gecko id	species
3	proportion of observations on each substrate	gecko id	substrate
4	proportion of observations on each substrate	gecko id	species*substrate

### Shear force as a measure of clinging performance

Gecko toepads are likely most adhesive after shedding, when lamellae, setae, and spatulae are intact and undamaged [92], therefore, clinging performance experiments were conducted within 3 days of shedding. Geckos were tested on coarse and fine sandpaper in a randomised order. We recorded the maximum shear force generated by a gecko’s toepads as outputs of the maximum force observed over the trial by attaching a force gauge (Extech 475,040; Extech equipment Pty Ltd., Australia) to the inguinal region of the gecko using a harness of fishing line with a diameter of 0.5 mm (Jarvis Walker Pty Ltd., Dandenong, Australia). Each gecko was allowed to take one step with each of the four feet on the testing substrate, thereby ensuring that the natural attachment system of the gecko was engaged [13, 93, 94]. Once each gecko made contact with all four feet, they were pulled horizontally backwards, at an angle of 0°, ensuring constant velocity (~ 0.5 cm per sec using a 30 cm-ruler and stopwatch), for 15 cm ( [45, 77, 94]; See [47] for details). Only one investigator (RP) conducted clinging performance trials to ensure consistency [21]. Each trial was repeated three times (*N* = 24) and five times (*N* = 8) on each substrate ( [49, 81, 90, 95], Supplementary material S3).

Clinging ability among species was compared using a set of 12 linear mixed-effects models in the R package *lme4* [88], which were compared using Akaike’s information criterion (AICc, [61]). Measures of shear force (three measures for 24 individuals and five measures for eight individuals) on each substrate were included as the response variable in all models. Substrate and species were included individually, additively, and as interactions in the candidate models (Table 5). Body size and toepad area are correlated [80, 86], with larger toepads more likely to have a greater area of setal fields that produce increased shear forces [10, 80, 96–98]. Hence, the attachment force generated by the adhesive system to a substrate should increase proportionally with an increase in toepad area and with mass ([75], Supplementary material S6). The three *Oedura* species we studied had different body sizes and toepad areas, therefore, to control for the influence of these variables on shear forces, we included toepad area and mass as fixed effects individually and additively. To account for inter-individual variation arising from repeated measures, we included individual gecko ID as a random factor in all candidate models. Shear force, mass and toepad area were log transformed prior to analyses (Table 5). Model selection was conducted using AICc for 12 candidate models using the R package *AICcmodavg* [89] to identify the model of best fit (ΔAICc < 2). Models with ΔAICc < 2, were averaged using the ‘model.avg’ function in the package *MuMIn* [99] and the relative importance of each variable in the averaged model was calculated. We conducted post hoc analyses on the best fit models (<2ΔAICc) to identify differences within the fixed effects using the R package *emmeans* [90]. Three measures of shear force, which exceeded three standard deviations from the mean of other measures, were identified as outliers and excluded from our analyses (*O. castelnaui* on coarse sandpaper: 1.23 N and 1.36 N; *O. castelnaui* on fine sandpaper: 1.76 N).

**Table 5:** Twelve candidate linear mixed-effects models used to analyse shear forces exerted by the three *Oedura* geckos, *O. castelnaui*, *O. monilis* and *O. coggeri*

Model	Response variable	Random effect	Fixed effects
1	log(shear force)	gecko ID	Substrate + log(toepad area)
2	log(shear force)	gecko ID	Substrate + log(mass)
3	log(shear force)	gecko ID	Substrate + log(mass) + log(toepad area)
4	log(shear force)	gecko ID	Substrate + Species + log(mass) + log(toepad area)
5	log(shear force)	gecko ID	Substrate + Species + log(mass)
6	log(shear force)	gecko ID	Substrate + Species + log (toepad area)
7	log(shear force)	gecko ID	Species + log(mass) + log(toepad area)
8	log(shear force)	gecko ID	Species + log(toepad area)
9	log(shear force)	gecko ID	Species + log(mass)
10	log(shear force)	gecko ID	Substrate*Species + log(toepad area)
11	log(shear force)	gecko ID	Substrate*Species + log(mass) + log(toepad area)
12	log(shear force)	gecko ID	Substrate*Species + log(mass)

## Supplementary information

**Additional file 1: Supplementary material S1.** Peak-to-valley heights (μm; Mean +/− SD) encountered by *Oedura* geckos in nature. **Supplementary material 2.** Microhabitat choice in *Oedura* geckos, *O. castelnaui, O. monilis* and *O. coggeri* including both vertical and horizontal observations*.* **Table S2.** Candidate models including both vertical and horizontal observations. Candidate models are arranged in increasing order of ΔAIC values and top model is in bold. Abbreviation: df, degrees of freedom. **Supplementary material S3.** Clinging performance performance in *Oedura* geckos. Mean shear force (Newton; Mean ± SE) from three trials (24 individuals) and five trials (eight individuals) on coarse and fine sandpaper. **Supplementary material S4**. Observations of microhabitat use of *Oedura* geckos in nature. **Supplementary material S5.** Substrate selection testing arena. **Supplementary material S6.** Relationship between mass (g) and shear force (N) on coarse (orange) and fine (blue) sandpaper in *Oedura* geckos.

## Data Availability

The datasets used and/or analysed during the current study are available from the corresponding author on reasonable request.
