# Supplementary material for: Geckos cling best to, and prefer to use, rough surfaces
Source: Front Zool. 2020 Oct 16;17:32. doi: 10.1186/s12983-020-00374-w (PMC7566132; doi:10.1186/s12983-020-00374-w)

**Clinging performance in relation to habitat preference and associated surface roughness**

**Supplementary material**

**Supplementary material S1.** Peak-to-valley heights (µm; Mean +/- SD) encountered by *Oedura* geckos in nature. Mean peak-to-valley heights were plotted against number of observations recorded on each microhabitat used by each of the three species, arboreal *O. castelnaui* (28 observations on dead trees and 40 observations on *E. melanophloia*), saxicoline, *O. coggeri* (17 observations on granite) and generalist, *O. monilis* (three observations on dead trees, seven observations on *E. melnaophloia*, six observations on *E. platyphylla*, four observations on *E. similis* and 20 observations on granite).


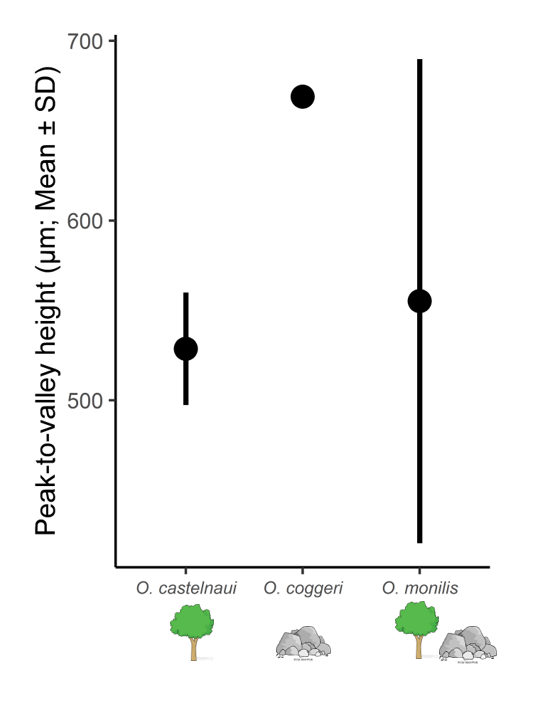


**Supplementary material 2.** Microhabitat choice in *Oedura* geckos, *O. castelnaui, O. monilis* and *O. coggeri* including both vertical and horizontal observations*.*

**Supplementary table 2.** Candidate models including both vertical and horizontal observations. Candidate models are arranged in increasing order of ΔAIC values and top model is in bold. Abbreviation: df, degrees of freedom.

| Fixed effects | ΔAIC | df | Weight | Residual Deviance |
| --- | --- | --- | --- | --- |
| **Substrate** | **0.0** | **5** | **0.64** | **2612.9** |
| Substrate + Species | 2.0 | 7 | 0.116 | 2610.9 |
| Species | 1298.5 | 4 | <0.001 | 2344.5 |

Post-hoc nalysis of microhabitat choice including both vertical and horizontal surfaces showed that *Oedura* geckos preffered coarse substrates compared to fine substrates (estimated marginal least square means *post-hoc* analysis, P < 0.01; Supplementary figure 2).


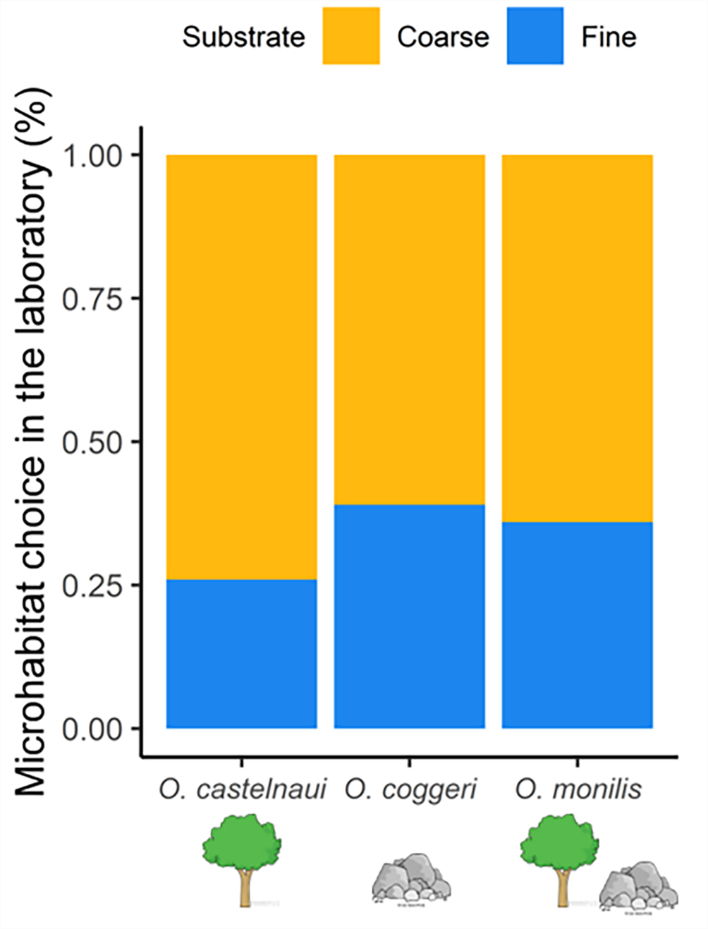


Supplementary figure 2

**Supplementary material S3.** Clinging performance performance in *Oedura* geckos. Mean shear force (Newton; Mean ±SE) from three trials (24 individuals) and five trials (eight individuals) on coarse and fine sandpaper.

| Species | Individual | Substrate | Number of trials | Shear force (Mean±SE) |
| --- | --- | --- | --- | --- |
| *O. castelnaui* | 1 | Coarse sandpaper | 3 | 0.84 ± 0.06 |
| *O. castelnaui* | 1 | Fine sandpaper | 3 | 0.04 ± 0.02 |
| *O. castelnaui* | 2 | Coarse sandpaper | 3 | 0.47 ± 0.02 |
| *O. castelnaui* | 2 | Fine sandpaper | 3 | 0.30 ± 0.07 |
| *O. castelnaui* | 3 | Coarse sandpaper | 3 | 0.80 ± 0.14 |
| *O. castelnaui* | 3 | Fine sandpaper | 3 | 0.87 ± 0.16 |
| *O. castelnaui* | 4 | Coarse sandpaper | 3 | 0.46 ± 0.09 |
| *O. castelnaui* | 4 | Fine sandpaper | 3 | 0.38 ± 0.11 |
| *O. castelnaui* | 5 | Coarse sandpaper | 3 | 0.80 ± 0.22 |
| *O. castelnaui* | 5 | Fine sandpaper | 3 | 0.20 ± 0.04 |
| *O. castelnaui* | 6 | Coarse sandpaper | 5 | 0.50 ± 0.06 |
| *O. castelnaui* | 6 | Fine sandpaper | 5 | 0.29 ± 0.03 |
| *O. castelnaui* | 7 | Coarse sandpaper | 3 | 0.42 ± 0.08 |
| *O. castelnaui* | 7 | Fine sandpaper | 3 | 0.50 ± 0.03 |
| *O. castelnaui* | 8 | Coarse sandpaper | 3 | 1.18 ± 0.12 |
| *O. castelnaui* | 8 | Fine sandpaper | 3 | 1.27 ± 0.27 |
| *O. castelnaui* | 9 | Coarse sandpaper | 5 | 0.71 ± 0.09 |
| *O. castelnaui* | 9 | Fine sandpaper | 5 | 0.71 ± 0.05 |
| *O. castelnaui* | 10 | Coarse sandpaper | 3 | 0.66 ± 0.02 |
| *O. castelnaui* | 10 | Fine sandpaper | 3 | 0.20 ± 0.03 |
| *O. coggeri* | 1 | Coarse sandpaper | 3 | 0.83 ± 0.03 |
| *O. coggeri* | 1 | Fine sandpaper | 3 | 0.23 ± 0.02 |
| *O. coggeri* | 2 | Coarse sandpaper | 3 | 0.46 ± 0.04 |
| *O. coggeri* | 2 | Fine sandpaper | 3 | 0.32 ± 0.02 |
| *O. coggeri* | 3 | Coarse sandpaper | 5 | 0.51 ± 0.06 |
| *O. coggeri* | 3 | Fine sandpaper | 5 | 0.32 ± 0.02 |
| *O. coggeri* | 4 | Coarse sandpaper | 3 | 0.61 ± 0.06 |
| *O. coggeri* | 4 | Fine sandpaper | 3 | 0.22 ± 0.04 |
| *O. coggeri* | 5 | Coarse sandpaper | 3 | 0.21 ± 0.02 |
| *O. coggeri* | 5 | Fine sandpaper | 3 | 0.16 ± 0.04 |
| *O. coggeri* | 6 | Coarse sandpaper | 3 | 0.86 ± 0.07 |
| *O. coggeri* | 6 | Fine sandpaper | 3 | 0.41 ± 0.04 |
| *O. coggeri* | 7 | Coarse sandpaper | 5 | 0.77 ± 0.12 |
| *O. coggeri* | 7 | Fine sandpaper | 5 | 0.42 ± 0.02 |
| *O. coggeri* | 8 | Coarse sandpaper | 5 | 0.66 ± 0.08 |
| *O. coggeri* | 8 | Fine sandpaper | 5 | 0.30 ± 0.05 |
| *O. coggeri* | 9 | Coarse sandpaper | 5 | 0.77 ± 0.12 |
| *O. coggeri* | 9 | Fine sandpaper | 5 | 0.42 ± 0.02 |
| *O. coggeri* | 10 | Coarse sandpaper | 5 | 0.56 ± 0.05 |
| *O. coggeri* | 10 | Fine sandpaper | 5 | 0.35 ± 0.06 |
| *O. coggeri* | 11 | Coarse sandpaper | 3 | 0.34 ± 0.02 |
| *O. coggeri* | 11 | Fine sandpaper | 3 | 0.22 ± 0.02 |
| *O. monilis* | 1 | Coarse sandpaper | 5 | 0.69 ± 0.07 |
| *O. monilis* | 1 | Fine sandpaper | 5 | 0.19 ± 0.02 |
| *O. monilis* | 2 | Coarse sandpaper | 3 | 1.01 ± 0.22 |
| *O. monilis* | 2 | Fine sandpaper | 3 | 1.10 ± 0.09 |
| *O. monilis* | 3 | Coarse sandpaper | 3 | 1.01 ± 0.07 |
| *O. monilis* | 3 | Fine sandpaper | 3 | 0.83 ± 0.07 |
| *O. monilis* | 4 | Coarse sandpaper | 3 | 1.38 ± 0.13 |
| *O. monilis* | 4 | Fine sandpaper | 3 | 0.61 ± 0.04 |
| *O. monilis* | 5 | Coarse sandpaper | 3 | 0.62 ± 0.03 |
| *O. monilis* | 5 | Fine sandpaper | 3 | 0.73 ± 0.12 |
| *O. monilis* | 6 | Coarse sandpaper | 3 | 0.45 ± 0.07 |
| *O. monilis* | 6 | Fine sandpaper | 3 | 0.37 ± 0.07 |
| *O. monilis* | 7 | Coarse sandpaper | 3 | 0.28 ± 0.06 |
| *O. monilis* | 7 | Fine sandpaper | 3 | 0.29 ± 0.04 |
| *O. monilis* | 8 | Coarse sandpaper | 3 | 1.46 ± 0.20 |
| *O. monilis* | 8 | Fine sandpaper | 3 | 0.84 ± 0.11 |
| *O. monilis* | 9 | Coarse sandpaper | 5 | 0.90 ± 0.22 |
| *O. monilis* | 9 | Fine sandpaper | 5 | 0.67 ± 0.11 |
| *O. monilis* | 10 | Coarse sandpaper | 3 | 1.13 ± 0.05 |
| *O. monilis* | 10 | Fine sandpaper | 3 | 1.43 ± 0.15 |
| *O. monilis* | 11 | Coarse sandpaper | 3 | 0.66 ± 0.15 |
| *O. monilis* | 11 | Fine sandpaper | 3 | 1.05 ± 0.05 |

**Supplementary material S4**. Observations of microhabitat use of *Oedura* geckos in nature.

| Species | Microhabitat | Locality | No. of observations | Date |
| --- | --- | --- | --- | --- |
| *O. castelnaui* | Dead tree | Cape York, QLD | 1 | Oct 2018 |
| *O. castelnaui* | Dead tree | Charters Towers Region, QLD | 27 | Sept 2015 – Feb 2017 |
| *O. castelnaui* | *E. melanophloia* | Townsville, QLD | 19 | May 2016 – Dec 2017;  June – July 2017 |
| *O. castelnaui* | *E. melanophloia* | Charters Towers Region, QLD | 21 | Aug 2015 – Feb 2017 |
| *O. monilis* | Dead tree | Collinsville, QLD | 3 | Nov 2019 |
| *O. monilis* | *E. melanophloia* | Collinsville, QLD | 6 | Nov 2019 |
| *O. monilis* | *E. melanophloia* | Hidden Valley, QLD | 1 | Aug 2017 |
| *O. monilis* | *E. platyphylla* | Collinsville, QLD | 6 | May 2020 |
| *O. monilis* | *E. similis* | Hidden Valley, QLD | 4 | Dec 2015; Aug 2017 |
| *O. monilis* | *Granite* | Cape Cleveland, QLD | 2 | December 2015 |
| *O. monilis* | *Granite* | Herveys Range, QLD | 1 | January 2015 |
| *O. monilis* | *Granite* | Hidden Valley, QLD | 10 | August 2017 |
| *O. monilis* | *Granite* | Magnetic Island, QLD | 7 | Oct – Nov 2015;  Jan 2018 |
| *O. coggeri* | *Granite* | Hidden Valley, QLD | 12 | Aug 2018 |
| *O. coggeri* | *Granite* | Undara, QLD | 5 | Nov 2017 |

**Supplementary material S5.** Substrate selection testing arena. Plastic containers (70 x 32 x 12 cm; Total area – 2448 cm2) were lined on all inner surfaces, with equal areas (1224cm2 of each) of 40 grit (coarse) and 400 grit (fine) aluminum oxide sandpaper. For video recording trials, testing arenas were covered with mesh, which was wrapped in transparent plastic film (Clorox Australia Pty Ltd, New South Wales, Australia) and sprayed with canola oil (Pascoe’s, Western Australia, Australia) to prevent the use of the mesh as a substrate by the geckos, while still allowing us to observe substrate choice. Surface on the left (blue) is fine sandpaper (P400) and right (grey) is coarse sandpaper (P40).


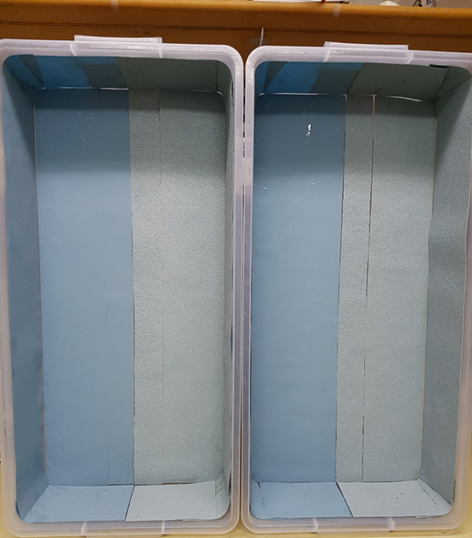


**Supplementary material S6.** Relationship between mass (g) and shear force (N) on coarse (orange) and fine (blue) sandpaper in *Oedura* geckos. (A) Relationship between mass and shear force on coarse and fine sandpaper in *O. castelnaui*. There was no significant influence of mass on shear force exerted by *O. castelnaui* (Linear model, df = 1, F = 2.10, P = 0.15) (B) Relationship between mass and shear force on coarse and fine sandpaper in *O. coggeri.* There was significant positive influence of mass on shear force in *O.* *coggeri* (Linear model, df = 1, F = 5.80, P < 0.05). (C) Relationship between mass and shear force on coarse and fine sandpaper in *O. monilis*. There was significant positive influence of mass on shear force in *O. monilis* (Linear model, df = 1, F = 14.54, P < 0.01).


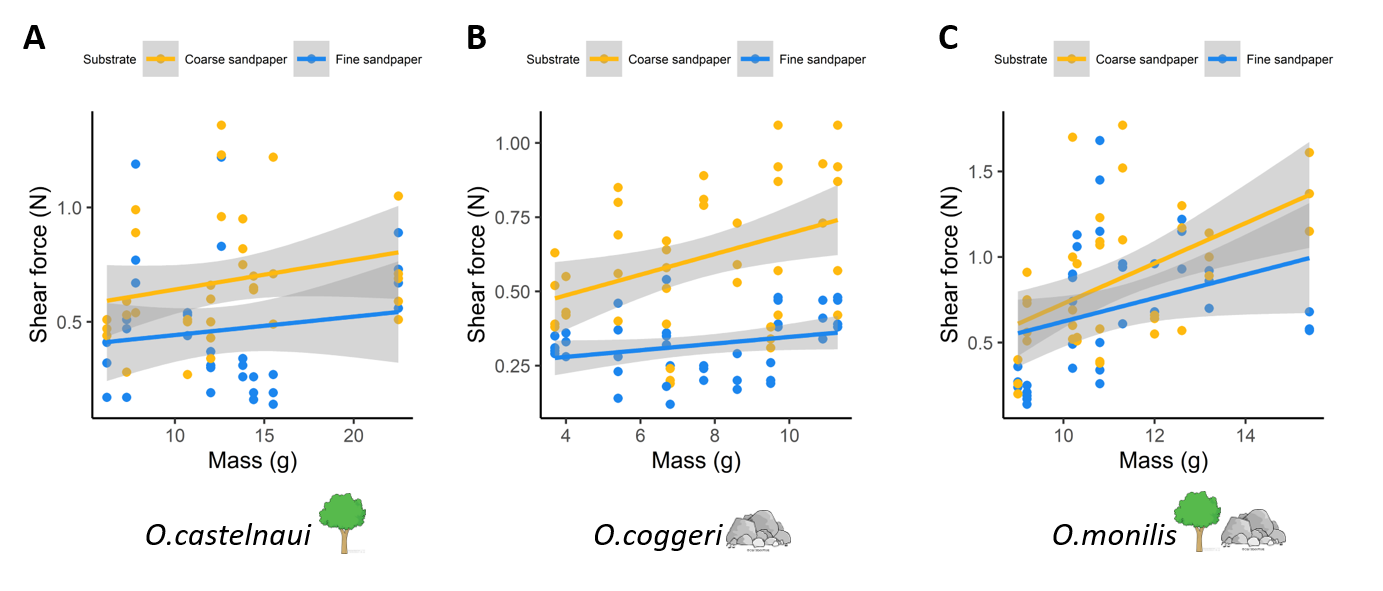

Supplement: Supplementary file 1 — Additional file 1: Supplementary material S1. Peak-to-valley heights (μm; Mean +/− SD) encountered by Oedura geckos in nature. Supplementary material 2. Microhabitat choice in Oedura geckos, O. castelnaui, O. monilis and O. coggeri including both vertical and horizontal observations. Table S2. Candidate models including both vertical and horizontal observations. Candidate models are arranged in increasing order of ΔAIC values and top model is in bold. Abbreviation: df, degrees of freedom. Supplementary material S3. Clinging performance performance in Oedura geckos. Mean shear force (Newton; Mean ± SE) from three trials (24 individuals) and five trials (eight individuals) on coarse and fine sandpaper. Supplementary material S4. Observations of microhabitat use of Oedura geckos in nature. Supplementary material S5. Substrate selection testing arena. Supplementary material S6. Relationship between mass (g) and shear force (N) on coarse (orange) and fine (blue) sandpaper in Oedura geckos. [file 12983_2020_374_MOESM1_ESM.docx]
